# Supplementary material for: Flat clathrin lattices are dynamic actin-controlled hubs for clathrin-mediated endocytosis and signalling of specific receptors
Source: Nat Commun. 2017 Jul 13;8:16068. doi: 10.1038/ncomms16068 (PMC5511353; doi:10.1038/ncomms16068)
Supplement: Supplementary Information [file ncomms16068-s1.pdf]

Type of file: PDF
Size of file: 0 KB
Title of file for HTML: Supplementary Information
Description: Supplementary Figures and Supplementary Table

Type of file: AVI
Size of file: 0 KB
Title of file for HTML: Supplementary Movie 1
Description: **Serum induces the dissolution of plaques.** TIRF movie of control KD cells expressing CLC-RFP before and after addition of 10% serum. Frame rate: 1 second.

Type of file: AVI
Size of file: 0 KB
Title of file for HTML: Supplementary Movie 2
Description: **Plaques are sites of CCV formation.** 3D SR stack of control KD cells stained for CHC. Scale bar, 1 µm.

Type of file: AVI
Size of file: 0 KB
Title of file for HTML: Supplementary Movie 3
Description: **LPAR1 is recruited to and internalized through clathrin-coated structures in control KD HeLa cells.** TIRF movie of control KD cells expressing CLC-RFP (red) and LPAR1-GFP (green) before and after stimulation with 5 µM LPA. Frame rate: 3 seconds.

Type of file: AVI
Size of file: 0 KB
Title of file for HTML: Supplementary Movie 4
Description: **LPAR1 is recruited to clathrin-coated structures but fails to be internalized in N-WASP KD HeLa cells.** TIRF movie of N-WASP KD cells expressing CLC-RFP (red) and LPAR1-GFP (green) before and after stimulation with 5 µM LPA. Frame rate: 3 seconds.

Type of file: PDF
Size of file: 0 KB
Title of file for HTML: Peer Review File
Description:

a **b**

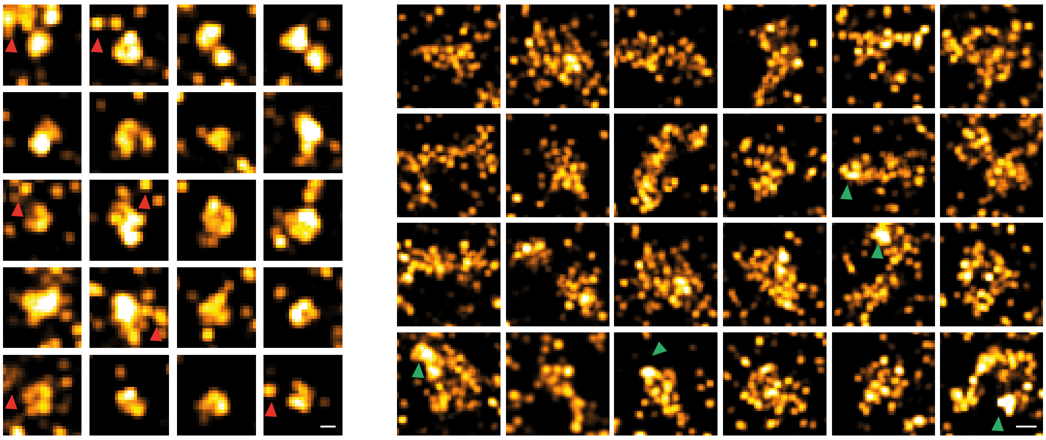

c

Clathrin Mediated Endocytosis	CME	Neuronal Wiskott-Aldrich Syndrome Protein	N-WASP
Clathrin Coated Vesicles	CCVs	Actin Related Protein 2/3 Complex	Arp2/3 Complex
Clathrin Coated Pits	CCPs, pits	verprolin, central, acidic	VCA
Flat Clathrin Plaques	FCPs, plaques	GTPase-Binding Domain	GBD
Clathrin Heavy Chain	CHC	Basic Region	B
Clathrin Light Chain	CLC	Proline-Rich Domain	PRD
Adaptor Protein Complex 2	AP2	WASP Homology Domain 1	WH1
Green Fluorescent Protein	GFP	WASP-Interacting Proteins	WIP
Knockdown	KD	Phosphatidylinositol 4,5-Biphosphate	PIP2
G-Protein Coupled Receptors	GPCR	Phosphatidylinositol 3,4,5 Trisphosphate	PIP3
Receptor Tyrosine Kinases	RTKs	Pleckstrin Homology	PH
Lysophosphatidic Acid	LPA	Electron Microscopy	EM
Epidermal Growth Factor	EGF	Total Internal Reflection Fluorescence	TIRF
EGF Receptor	EGFR	Correlative Light And Electron Microscopy	CLEM
LPA Receptor 1	LPAR1	Super-Resolution Microscopy	SR
LPA Receptor 3	LPAR3	Ground State Depletion followed by Individual Molecule Return	GSDIM

Leyton-Puig et. al. Supplementary Figure 1

Supplementary Figure 1. Gallery of representative CCSs observed at the basal membrane in HeLa cells.

(**a**) Pits are homogenous circular structures. Representative SR images of pits obtained from control KD and N-WASP KD HeLa cells stained for CHC. Scale bar, 100 nm. Red arrowheads mark structures that were not classified as pits. (**b**) Plaques are heterogeneous structures. Representative SR images of plaques obtained from control KD and N-WASP KD HeLa cells stained for CHC. Green arrowheads mark pits or vesicles in the periphery of plaques. Scale bar, 500 nm. (**c**) Table listing the abbreviations used in this manuscript.

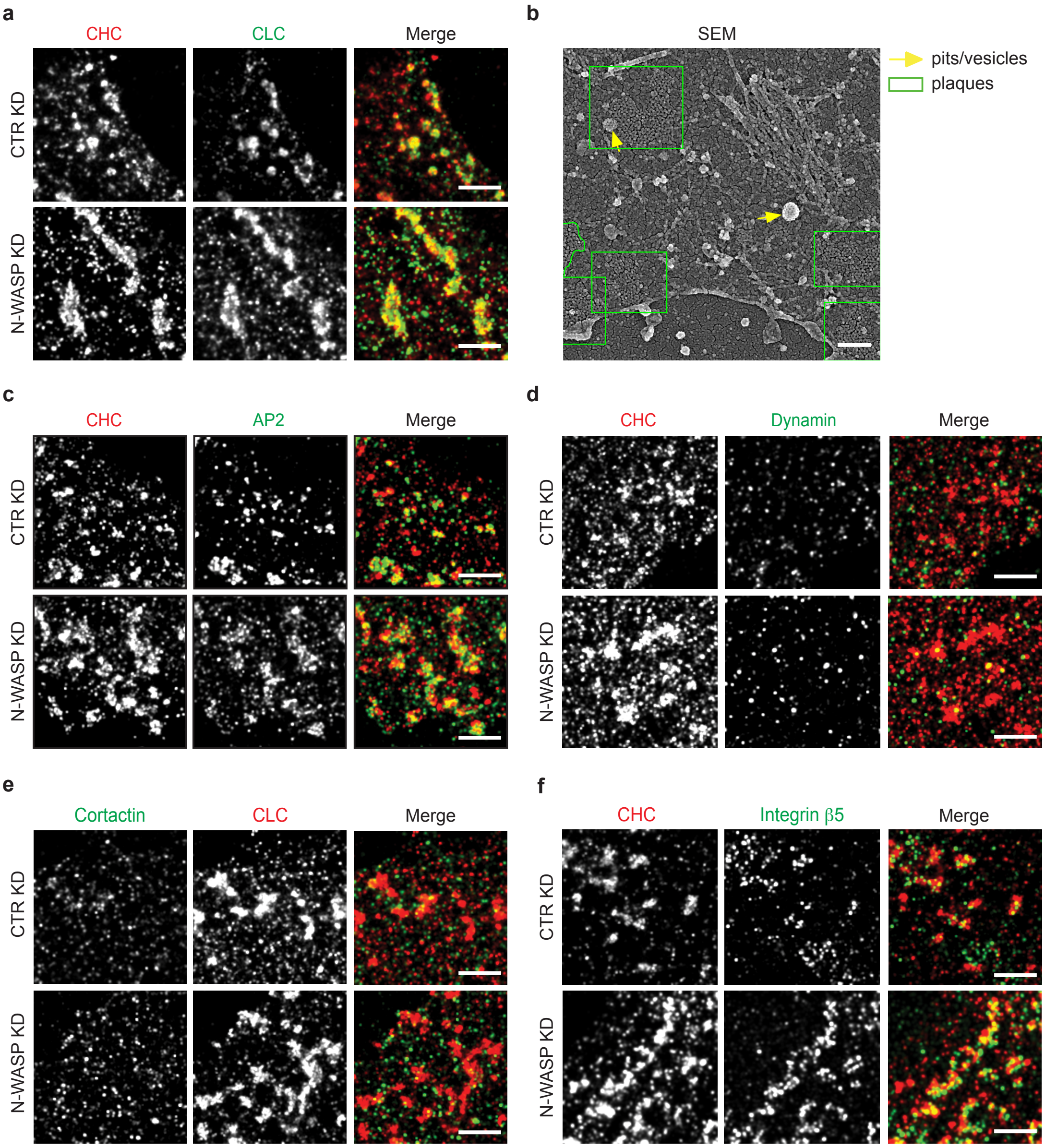

Leyton-Puig et. al. Supplementary Figure 2

Supplementary Figure 2. Molecular characterization of pits and plaques

(**a**) Clathrin light and heavy chain colocalize in CCSs. Representative two-color SR images of control (CTR) KD and N-WASP KD HeLa cells expressing CLC-mTQ2 and stained for GFP (green in merge) and CHC (red in merge). Scale bar, 1 µm. (**b**) Scanning electron microscopy (SEM) shows pits and plaques in the N-WASP KD cells. Representative SEM picture shows pits and plaques highlighted by yellow arrows and green boxes, respectively. Scale bar, 300 nm. (**c**) CHC and AP2 colocalize in CCSs. Representative two-color SR images of control (CTR) KD and N-WASP KD cells expressing AP50-GFP and stained for CHC (red in merge) and GFP (green in merge). Scale bar, 1 µm. (**d**) Dynamin localizes in pits and plaques. Representative two-color SR images of control (CTR) KD and N-WASP KD cells expressing Dynamin2-GFP and stained for CHC (red in merge) and GFP (green in merge). Scale bar, 1 µm. (**e**) Cortactin localizes in pits and plaques. Representative two-color SR images of control (CTR) KD and N-WASP KD cells expressing CLC-mTQ2 and stained for Cortactin (green in merge) and GFP (red in merge). Scale bar, 1 µm. (**f**) Integrin β5 localizes in pits and plaques. Representative two-color SR images of control (CTR) KD and N-WASP KD cells stained for CHC (red in merge) and Integrin β5 (green in merge). Scale bar, 1 µm.

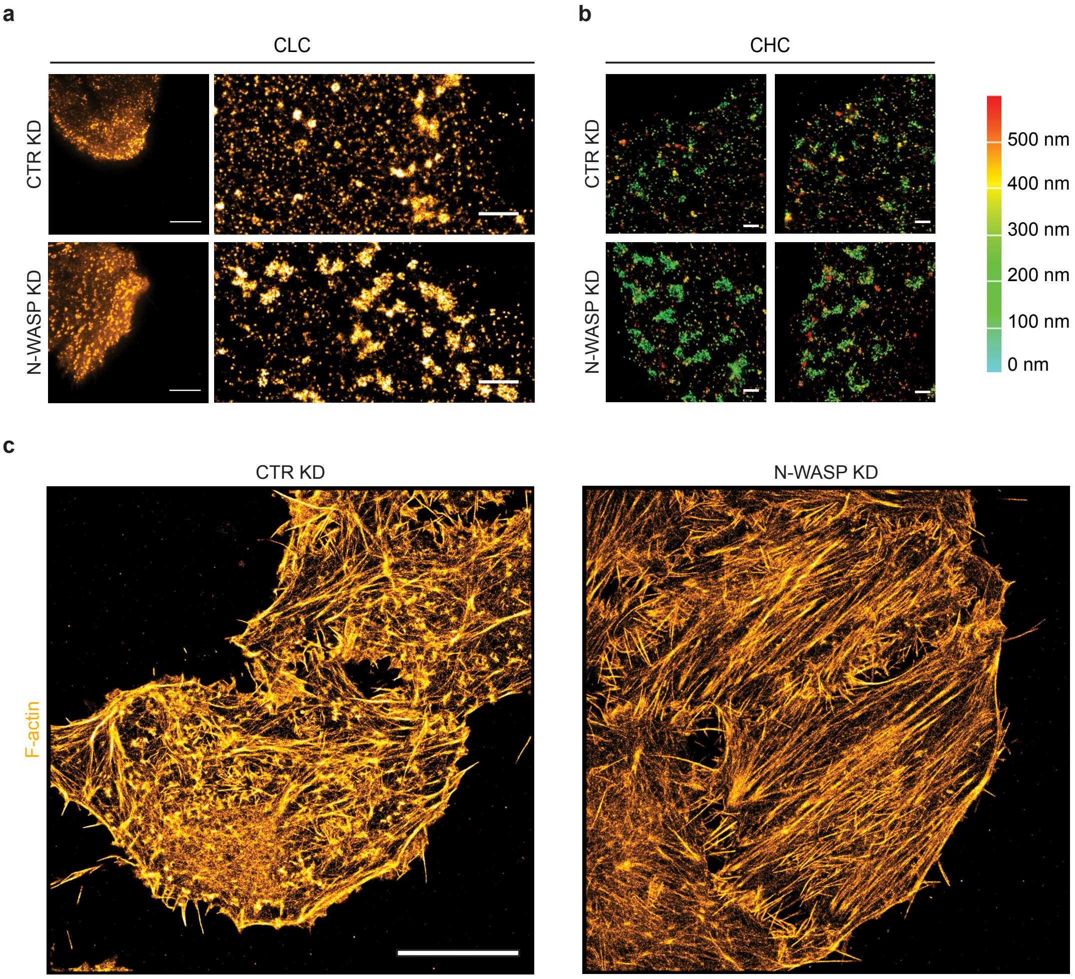

Leyton-Puig et. al. Supplementary Figure 3

Supplementary Figure 3. Knockdown of N-WASP in HeLa cells results in abundant plaques located close to the basal membrane.

(**a**) Representative TIRF and SR images of control (CTR) KD and N-WASP KD HeLa cells expressing CLC-mTQ2 and stained for GFP. Scale bar TIRF, 10 µm. Scale bar SR images, 1 µm. (**b**) Knockdown of N-WASP increases the presence of plaques close to the basal membrane. Representative projections of 3D SR images, color-coded for depth, of control (CTR) KD and N-WASP KD cells stained for CHC. Scale bar, 1 µm. (**c**) Knockdown of N-WASP increases basal actin stress fibers. Representative SR images of control (CTR) KD and N-WASP KD cells stained with phalloidin to detect F-actin. Scale bar, 10 µm.

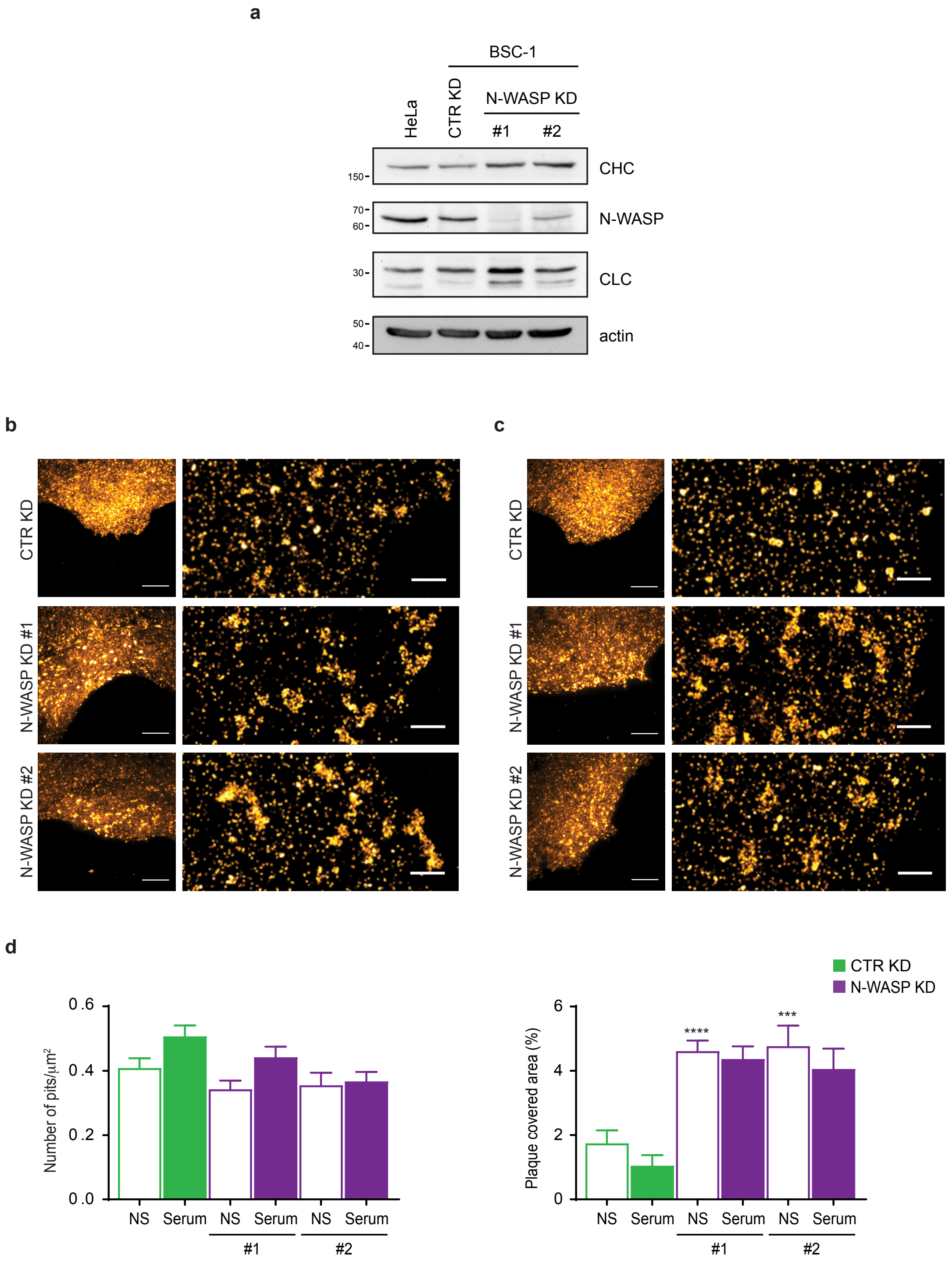

Leyton-Puig et. al. Supplementary Figure 4

Supplementary Figure 4. N-WASP is involved in plaque regulation in BSC-1 cells.

(**a**) Characterization of control (CTR) KD and N-WASP KD (#1 and #2 obtained using different hairpins) BSC-1 cells. Total cell lysates were compared using the indicated antibodies. One of two experiments that were performed with similar results is shown. (**b**) Knockdown of N-WASP induces plaque presence in the plasma membrane. Representative TIRF and SR images of control (CTR) KD and N-WASP KD (#1 and #2 obtained using different hairpins) BSC-1 cells that were serum deprived overnight and stained for CHC. Scale bar TIRF images, 10 μm. Scale bar SR images, 1 μm. (**c**) Knockdown of N-WASP makes plaques insensitive to serum. Representative TIRF and SR images of growing control (CTR) KD and N-WASP KD (#1 and #2) BSC-1 cells stained for CHC. Scale bar TIRF images, 10 μm. Scale bar SR images, 1 μm. (**d**) Bar graph shows number of pits per μm^2 and percentage of area covered by plaques in cells that were serum starved overnight or kept in growing conditions (Serum) (mean +/- SEM, *** $P<0.001$, **** $P<0.0001$, one-way ANOVA; n = 21 cells control NS, n = 14 control cells serum, n = 20 cells N-WASP KD #1 NS, n = 21 cells N-WASP KD #1 serum, n = 12 cells N-WASP KD #2 NS, n = 11 cells N-WASP KD #2 serum, pooled from three independent experiments).

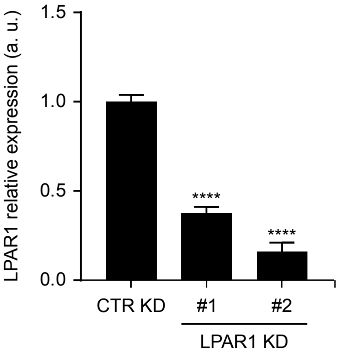

Leyton-Puig et. al. Supplementary Figure 5

Supplementary Figure 5. Validation of LPAR1 knockdown cells.

Stable control (CTR) KD and LPAR1 KD (#1 and #2 obtained using different hairpins) HeLa cells were obtained as described in the Methods. Total RNA extraction and RT-qPCR analysis were done as described in the Methods. Bar graph shows mean +/- SEM of relative mRNA levels of LPAR1 (n = 3; **** P<0.0001, one-way ANOVA). Data are normalized with respect to the middle value obtained from the control KD cells using GADPH as housekeeping gene.

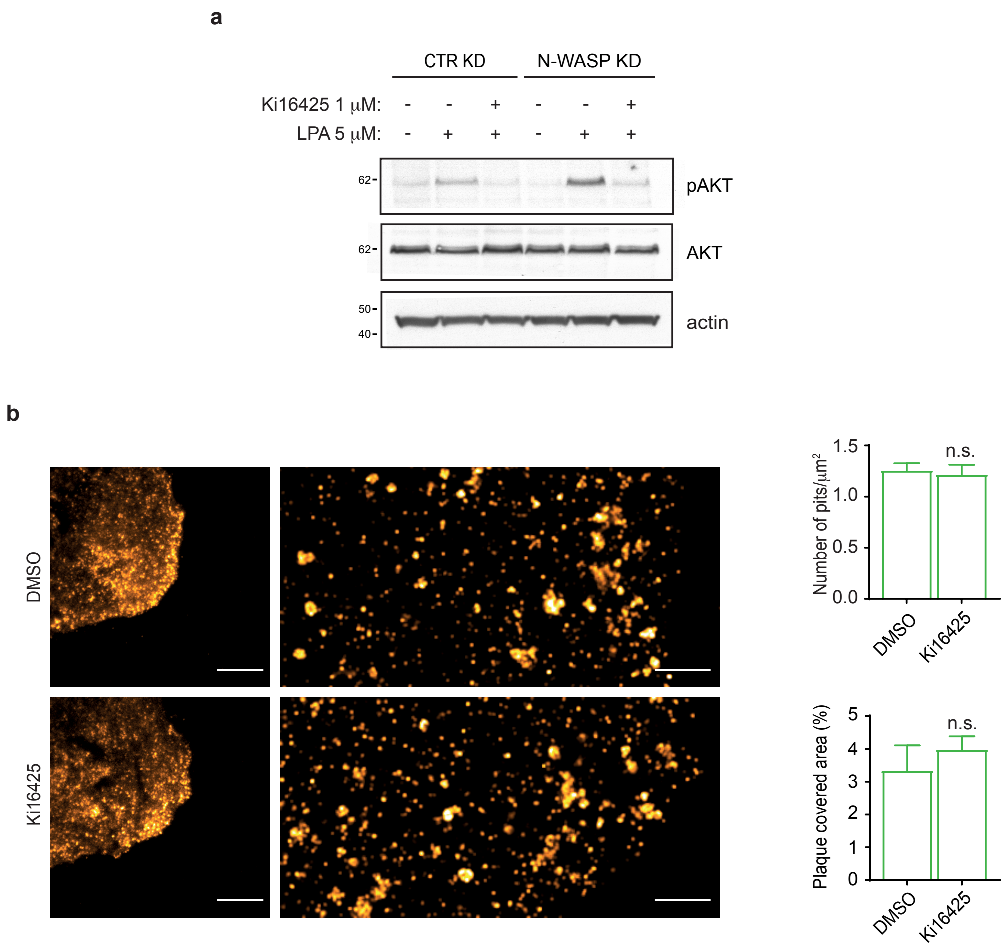

Leyton-Puig et. al. Supplementary Figure 6

Supplementary Figure 6. AKT hyper-activation in N-WASP KD cells is mediated by LPAR1.

(**a**) Western blot analysis of AKT activation in control (CTR) KD and N-WASP KD HeLa cells after stimulation with 5 µM LPA for 5 minutes with and without preincubation with the LPA receptor inhibitor Ki16425. Total cell lysates were compared using the indicated antibodies. One of three experiments that were performed with similar results is shown. (**b**) Ki16425 does not perturb basal CCSs in HeLa cells. Representative TIRF and SR images of basal CCSs found in serum-starved control KD cells treated with vehicle (DMSO) or the LPA receptor inhibitor Ki16425 (5 µM) for 5 minutes and stained for CHC. Scale bar TIRF images, 10 µm; SR images, 1 µm. Bar graphs show the number of pits per μm^2 and percentage of total area of the ROI covered by plaques as mean +/- SEM (n = 15 cells for ethanol, n = 16 cells for Ki16425, pooled from two independent experiments).

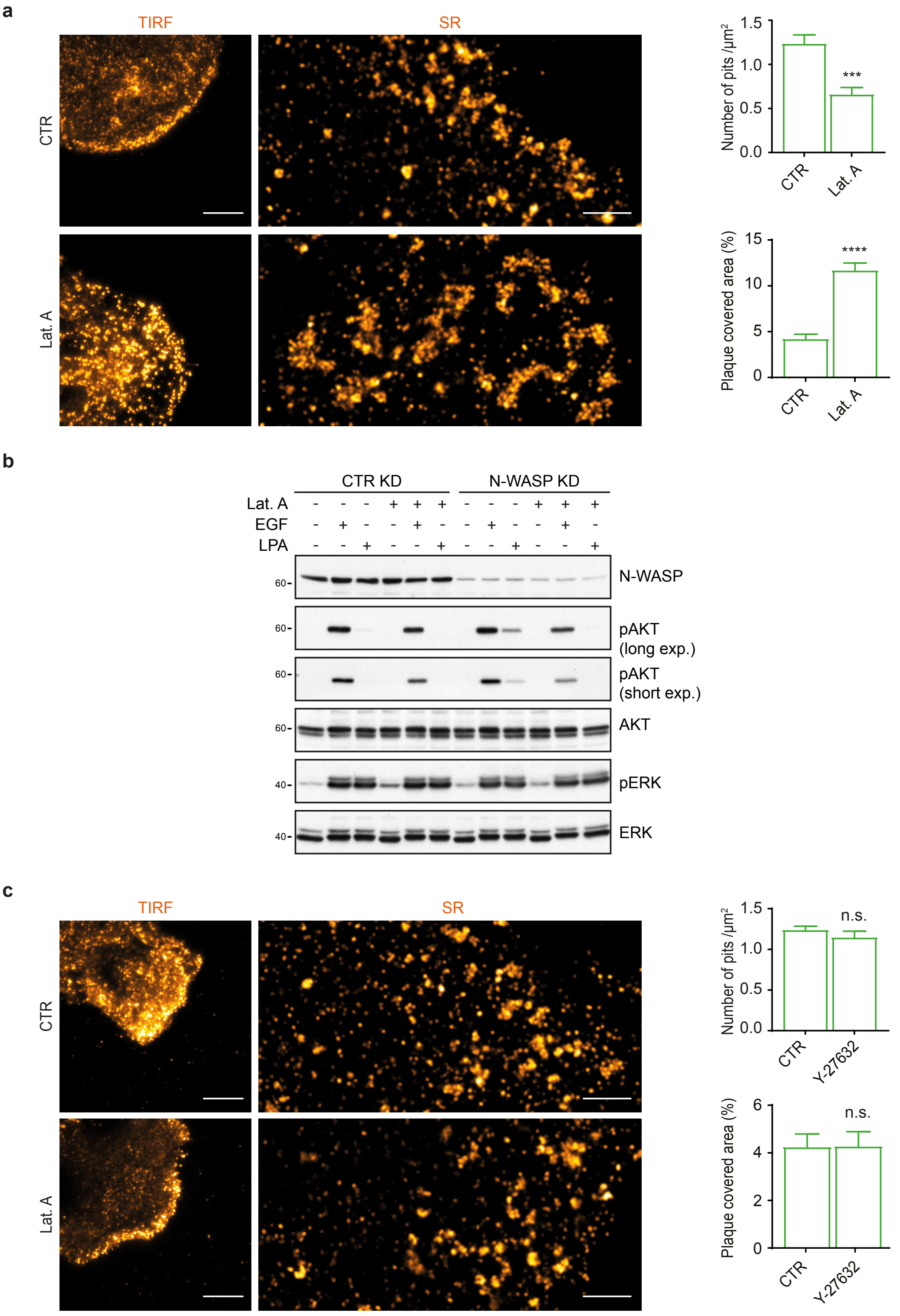

Leyton-Puig et. al. Supplementary Figure 7

Supplementary Figure 7. Plaque formation does not scale with acto-myosin tension.

(**a**) Latrunculin A perturbs both pits and plaques. Representative TIRF and SR images of CCSs on the basal membrane of HeLa cells. Control KD HeLa cells were treated with either Latrunculin A (Lat. A, 0.25 μM) or ethanol as a control (CTR) for 15 minutes, fixed and stained with anti-clathrin heavy chain (CHC) antibodies as indicated in the Methods. Scale bar TIRF images, 10 μm; SR images, 1 μm. Bar graphs show the number of pits per μm^2 and percentage of total area of the ROI covered by plaques (mean +/- SEM, *** $p < 0.001$, **** $p < 0.0001$, t-test; n =13 cells for ethanol, n= 13 cells for Latrunculin A, pooled from three independent experiments). (**b**) Latrunculin A inhibits LPA-induced and EGF-induced activation of AKT independently of N-WASP expression. Western blot analysis of AKT and ERK activation in control (CTR) KD and N-WASP KD cells pretreated with either Latrunculin A (Lat. A, 0.25 μM) or vehicle (-, ethanol) for 15 minutes prior to stimulation with either EGF (100 ng/ml) or LPA (5 μM) for 5 minutes. Total cell lysates were compared using the indicated antibodies (long exp. = long exposure, short exp. = short exposure). One of two experiments that were performed with similar results is shown. (**c**) Inhibition of ROCK does not affect either pits or plaques. Control KD HeLa cells were treated with either Y27632 or DMSO as a control (CTR) for 15 minutes, fixed and stained as above. Scale bar TIRF images, 10 μm; SR images, 1 μm. Bar graphs show mean +/- SEM of the number of pits per μm^2 and percentage of total area of the ROI covered by plaques (n = 16 cells for DMSO, n= 16 cells for Y27632, pooled from two independent experiments).

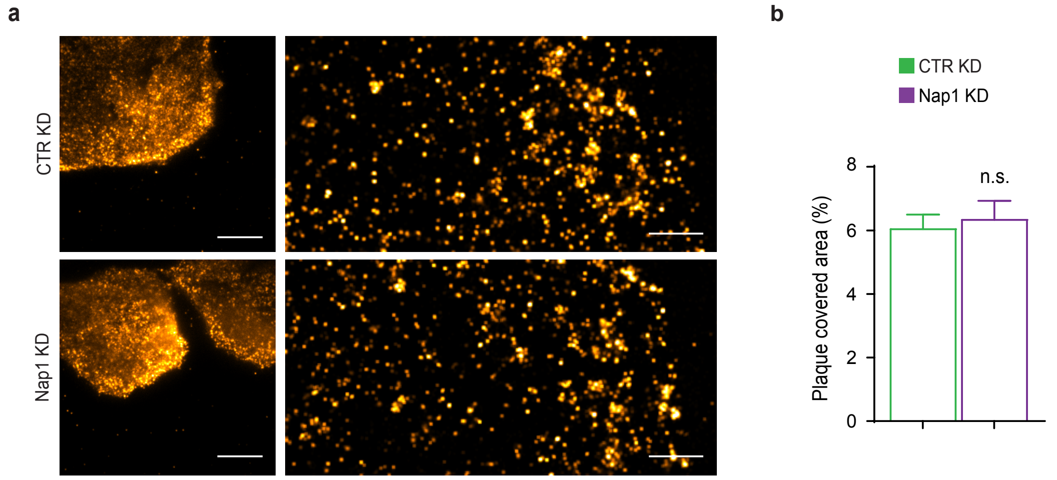

Leyton-Puig et. al. Sup Figure 8

Supplementary Figure 8. Downregulation of Abi1 in Nap1 KD cells does not affect plaques.

(**a**) Representative TIRF and SR images of basal CCSs found in serum-starved control (CTR) KD and Nap1 KD cells stained for CHC. Scale bar TIRF images, 10 µm; SR images, 1 µm. (**b**) Bar graphs show percentage of total area of the ROI covered by plaques in starved control (CTR) KD and Nap1 KD cells (mean +/- SEM, n = 15 cells CTR KD, n = 15 cells Nap1 KD, pooled from three independent experiments).

a

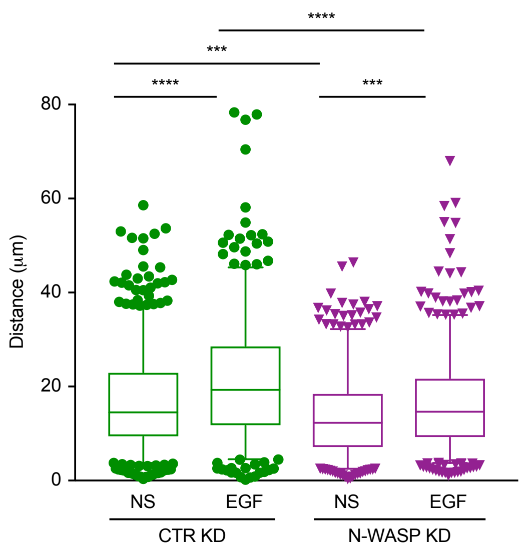

b

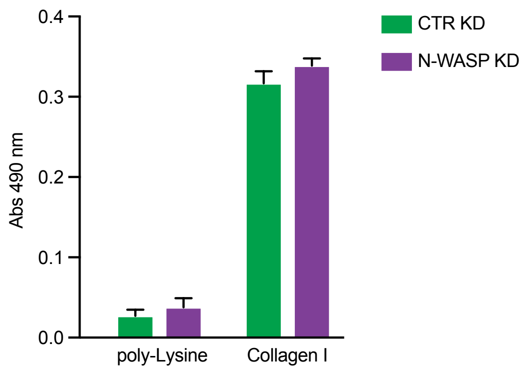

Leyton-Puig et. al. Supplementary Figure 9

Supplementary Figure 9. Increased plaque abundance inversely correlates with cell migration and does not affect cell adhesion.

(**a**) Plaque abundance inversely correlates with random cell migration. Control (CTR) KD and N-WASP KD cells were plated on gelatine-coated plates, serum starved overnight, stimulated with EGF (100 ng/ml) or left untreated and subsequently imaged as described in the Methods. Box plot shows median displacement in micrometers (μm) and 5-95 percentiles (control KD cells: n = 621 NS, n = 413 EGF; N-WASP KD cells: n = 473 NS, n = 563 EGF, pooled from two independent experiments). (**b**) Plaque abundance does not affect either integrin-independent or integrin-dependent cell adhesion. Control (CTR) KD and N-WASP KD cells were detached and seeded to probe integrin-independent and integrin-dependent cell adhesion on poly-Lysine and Collagen I, respectively, as described in the Methods. Bar graph shows mean +/- SEM (n = 10, pulled from two independent experiments).

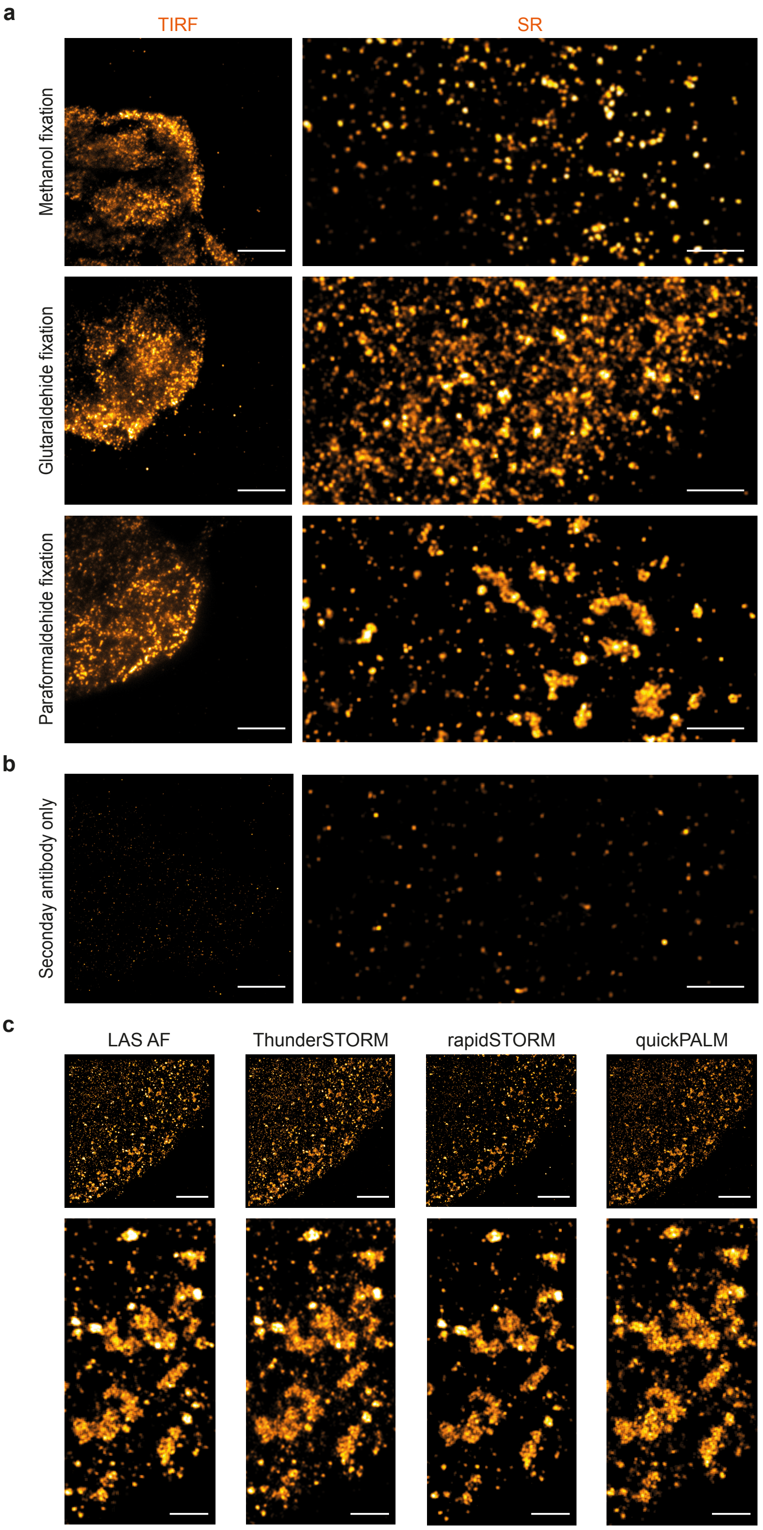

Leyton-Puig et. al. Supplementary Figure 10

Supplementary Figure 10. Fixation procedures, but not localization analysis algorithms, affect presence and shape of pits and plaques in SR images.

(**a**) Fixative strongly affects the outcome of SR images. Representative TIRF and SR images of CHC on the basal membrane of control KD cells fixed with Methanol, Glutaraldehyde or Paraformaldehyde. Scale bar TIRF images, 10 µm; SR images, 1 µm. (**b**) Secondary antibody-only staining results in SR images having few random localizations. Representative SR image and blow-up of the basal membrane of control KD cells stained only with secondary anti mouse antibodies. Scale bar 10 µm; SR blow-up, 1 µm. (**c**) CCS shape and distribution are not affected by the choice of SR localization algorithm. SR images of CHC-positive structures on the basal membrane of control KD cells were analyzed with the localization algorithm LAS AF from Leica Microsystems, ThunderSTORM, rapidSTORM and quickPALM. Scale bar SR 5 µm; blow-up, 1 µm.

Fig. 2a

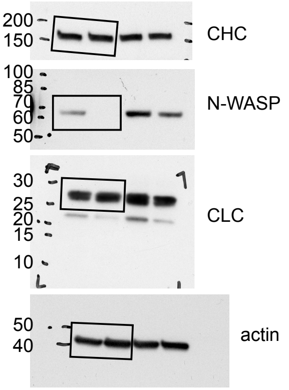

Fig. 3g

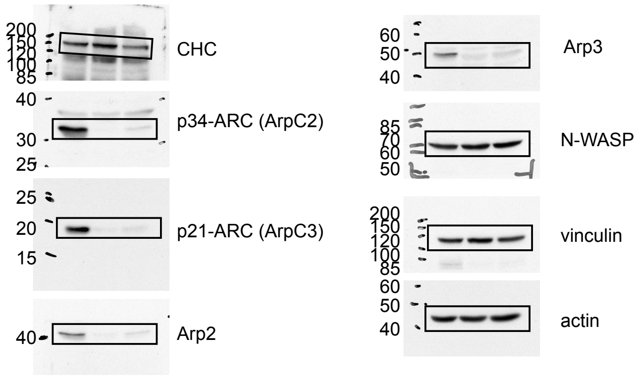

Fig. 7b

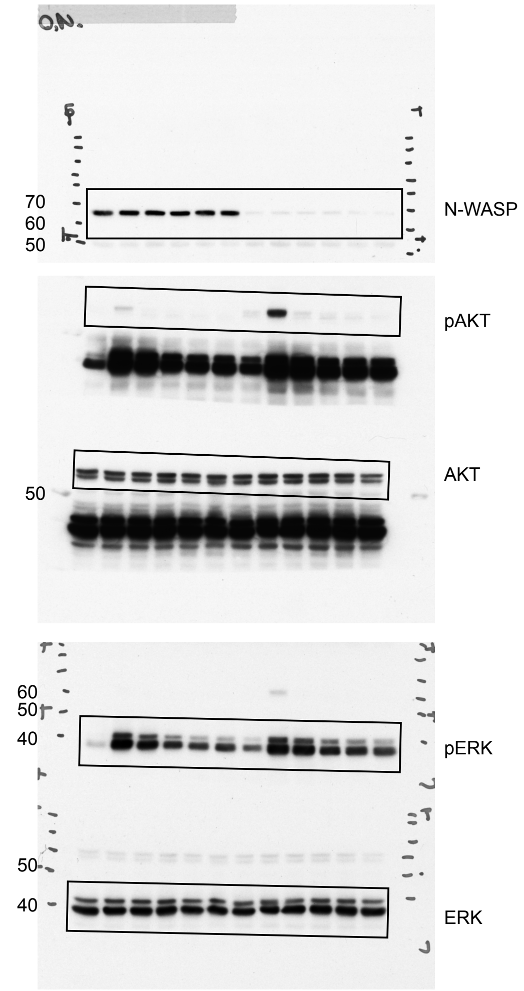

Supplementary Fig. 4a

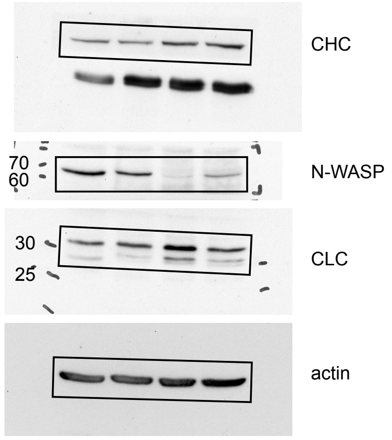

Supplementary Fig. 6a

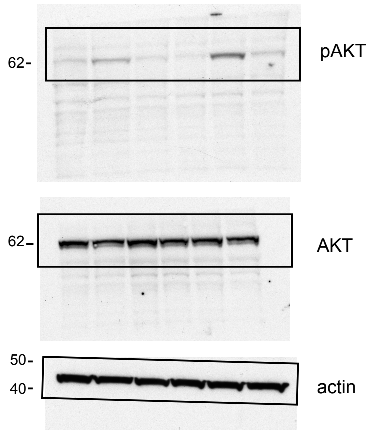

Leyton-Puig et. al. Supplementary Figure 11

Supplementary Figure 11. Uncropped scans of the most important blots.

Boxes indicate cropped regions.

Supplementary Table 1. PCR primers.

Primer name	Primer sequence (5' - 3')
ratN-WASP H208D Fwd	GTAATTTCCAGgacATTGGACAT
ratN-WASP H208D Rev	TTGGTGTTCCAATATCTGCCT
